# *In vitro* stability of therapeutically relevant, internally truncated dystrophins

**DOI:** 10.1186/s13395-015-0040-z

**Published:** 2015-04-28

**Authors:** Jackie L McCourt, Katrina K Rhett, Michele A Jaeger, Joseph J Belanto, Dana M Talsness, James M Ervasti

**Affiliations:** Department of Biochemistry, Molecular Biology, and Biophysics, University of Minnesota - Twin Cities, Minneapolis, MN 55455 USA

**Keywords:** Duchenne muscular dystrophy, Becker muscular dystrophy, Dystrophin, Utrophin, Exon skipping, Gene therapy

## Abstract

**Background:**

The X-linked recessive disease Duchenne muscular dystrophy (DMD) is caused by mutations in the gene encoding the protein dystrophin. Despite its large size, dystrophin is a highly stable protein, demonstrating cooperative unfolding during thermal denaturation as monitored by circular dichroism spectroscopy. In contrast, internal sequence deletions have been associated with a loss of the cooperative unfolding and cause *in vitro* protein aggregation. Several emerging therapy options for DMD utilize internally deleted micro-dystrophins and multi-exon-skipped dystrophins that produce partially functional proteins, but the stability of such internally truncated proteins has not been investigated.

**Methods:**

In this study, we analyzed the *in vitro* stability of human dystrophin constructs skipped around exon 45 or exon 51, several dystrophin gene therapy constructs, as well as human full-length and micro-utrophin. Constructs were expressed in insect cells using the baculovirus system, purified by affinity chromatography, and analyzed by high-speed sedimentation, circular dichroism spectroscopy, and differential scanning fluorimetry.

**Results:**

Our results reveal that not all gene therapy constructs display stabilities consistent with full-length human dystrophin. However, all dystrophins skipped in-frame around exon 45 or exon 51 show stability profiles congruent with intact human dystrophin. Similar to previous studies of mouse proteins, full-length human utrophin also displays stability similar to human dystrophin and does not appear to be affected by a large internal deletion.

**Conclusions:**

Our results suggest that the *in vitro* stability of human dystrophin is less sensitive to smaller deletions at natural exon boundaries than larger, more complex deletions present in some gene therapy constructs.

## Background

The X-linked disease Duchenne muscular dystrophy (DMD) is caused by mutations in the gene encoding the protein dystrophin [1]. Mutations causing this disease are variable with 65% of DMD patients harboring deletions which span exons, 5% to 15% having duplications, and the remaining populations having either point mutations or deep intronic deletions [2]. Becker muscular dystrophy (BMD) is a milder allelic form of dystrophy typically caused by in-frame gene deletions that maintain reading frame but presumably cause disease through diminished abundance or functionality [3].

The dystrophin protein is a critical molecular component of the dystrophin-glycoprotein complex (DGC) that functions to maintain skeletal muscle integrity during contraction [4,5]. Dystrophin provides a structural link between the sub-sarcolemmal cytoskeleton and the extracellular matrix through interactions with actin [6,7], intermediate filaments [8-10], microtubules [11,12], and the membrane-associated dystroglycan complex [13,14]. The observation that milder BMD patients harbor deletions in the central rod domain suggests that dystrophin can tolerate such deletions to some degree and that the central rod domain is less critical to the function of dystrophin.

Two avenues of therapeutic research have focused on producing internally truncated, Becker-like dystrophins in DMD patients. Exon-skipping approaches aim to restore the reading frame of mutated DMD transcripts using antisense oligonucleotides (ASOs) or phosphorodiamidate morpholino oligomers (PMOs), producing an internally truncated but partially functional protein [15-17]. Alternatively, adeno-associated viral (AAV) gene therapy is under active investigation to express miniaturized dystrophin constructs in DMD patients due to the large size of the dystrophin gene and the limited capacity of AAV vectors [18-20].

The stability of the corresponding proteins produced from dystrophin exon skipping or AAV-mediated delivery of micro-dystrophins is unknown and may be an important factor to maximize therapeutic efficacy. Previous *in vitro* work has demonstrated that the stability of mouse dystrophin was sensitive to disease-causing missense mutations and internal deletion [21,22], raising the question of whether the stabilities of micro-dystrophins or exon-skipped dystrophins relevant to DMD therapies might also be compromised. In contrast, the stability of mouse utrophin, a fetal homologue of dystrophin, was insensitive to both terminal and internal deletion [22].

Here, we expressed and purified five dystrophins skipped around exon 45 or 51 with an exon-43-skipped control, five recombinant dystrophin gene therapy constructs, and two utrophin constructs. The selected constructs represent the leading therapy approaches that have been shown to ameliorate the dystrophic phenotype in *mdx* mice with transition to clinical trials underway [19,20,23-28]. In the current study, all constructs were expressed from human sequences, as opposed to the mouse constructs used previously [21,22]. Our biophysical analysis revealed that the dystrophin gene therapy constructs exhibited more variable stabilities *in vitro* while exon-skipped dystrophin constructs showed stabilities not different from full-length dystrophin. Consistent with previous mouse studies, utrophin maintained stability despite internal deletion.

## Methods

### Cloning

A cDNA corresponding to the sequence of full-length human dystrophin was obtained from the DNASU vector repository in the pE223 Gateway entry vector. Human utrophin and micro-utrophin were cloned from HEK293 cells into the pENTR/D-TOPO vector (Invitrogen™, Waltham, MA, USA) and sequence verified. An eight-amino-acid FLAG-tag (DYKDDDDK) was added to the N-terminus of both human dystrophin and utrophin constructs for use in purification. All human dystrophin deletion constructs were PCR amplified using primers designed around adjacent exons, repeats, or domains for the desired deletion based on reported repeat and domain boundaries [29]. The PCR products were circularized using T4 polynucleotide kinase and T4 DNA ligase (New England BioLabs, Ipswich, MA, USA) and sequence verified. Using the Gateway Recombination system (Life Technologies, Carlsbad, CA, USA), the deletion constructs were recombined into the pDEST8 destination vector and subsequently transformed into DH10Bac-competent *Escherichia coli* and purified according to the manufacturer’s protocol.

### Protein expression and purification

Sf9 insect cells were maintained at 1 × 10^6^ cells/mL in Sf-900™ II SFM (Life Technologies, Carlsbad, CA, USA). Purified baculovirus was transfected using Cellfectin® II (Life Technologies, Carlsbad, CA, USA), and high-titer viral stocks were generated through successive infections of Sf9 cells in 3.5-cm plates (P0), 10-cm plates (P1), and 250 mL of 1 × 10^6^ cells/mL suspended cells (P2). Ten milliliters of P2 virus was used to infect 250 mL of 1 × 10^6^ cells/mL and cultured for 72 h post-infection to maximize protein expression. Infected cells were centrifuged at 1,000 × *g* for 3 min and re-suspended in phosphate-buffered saline (PBS) containing a cocktail of protease inhibitors (100 nM Aprotinin, 10 mg/mL E-64, 10 μM Leupeptin, 1 mM PMSF, 1 μg/mL Pepstatin). Cells were lysed by sonication, five bursts of 30 s using a BioLogics Ultrasonic Homogenizer set at 30% power. The lysate was centrifuged at 14,000 × *g* for 10 min at 4°C and the supernatant applied to an anti-FLAG M2 agarose column (Sigma Aldrich, St. Louis, MO, USA). The column was washed with >10 column volumes of PBS and bound protein eluted with PBS-containing protease inhibitors and 100 μg/mL FLAG peptide. After dialysis overnight in 2 L of PBS at pH 7.5, the purified protein was concentrated using the Amicon Centrifugal Filter unit (UFC801024), and protein concentration was determined using A_280_ and extinction coefficients calculated from the amino acid compositions for each construct. Concentrated proteins were run on a 3% to 12% sodium dodecyl sulfate (SDS) polyacrylamide gradient gel and run at 150 V for 1 h. Gels were stained with Coomassie blue stain and visualized using Licor’s Odyssey® Infrared Imaging System.

### Tandem purification of full-length human dystrophin

To optimize the purification of full-length human dystrophin, a Twin-Strep-tag® (IBA Life Sciences, Göttingen, Germany), with amino acid sequence SA-WSHPQFEK(GGGS)_2_GGSAWSHPQFEK, was cloned onto the C-terminus of pE223 dystrophin in addition to the N-terminal FLAG-tag. The dual-tagged dystrophin was then recombined into the pDEST8 expression vector and expressed in the *Sf9* baculovirus system as described above. The cell lysate was applied to a *Strep*-Tactin ® Superflow ® high-capacity resin (IBA Life Sciences, Göttingen, Germany), eluted with 100 mM Tris-HCl, 150 mM NaCl, 1 mM EDTA, 2 mM desthiobiotin, pH 8, and the eluent immediately applied to an anti-FLAG M2 agarose column as described above.

### Western blotting

Purification fractions from the tandem purification were run on a 3% to 12% SDS polyacrylamide gradient gel at 150 V for 1 h. The gel was transferred onto a polyvinylidene difluoride (PVDF) membrane at 100 V for 1 h. The PVDF membrane was blocked using 5% milk in 1X PBS and 0.1% Tween and blotted using mouse monoclonal anti-FLAG M2 (F1804, Sigma Aldrich, St. Louis, MO, USA) and rabbit polyclonal anti-Strep-tag II (ab76949, Abcam, Cambridge, UK) antibodies at 1:1,000 dilution. The blot was visualized using anti-mouse DyLight™ 800 (green channel) and anti-rabbit DyLight™ 680 (red channel) conjugated antibodies in Licor’s Odyssey® Infrared Imaging System.

### High-speed sedimentation

Each purified protein was diluted to 0.3 mg/mL (for exon-51-skipped dystrophins) or 0.5 mg/mL (for all other proteins) with PBS in a final volume of 120 μL, and 60 μL was immediately aliquoted into 12 μL 6X Laemmli sample buffer (LSB) to prepare a ‘total’ fraction. The remaining 60 μL was centrifuged at 100,000 × *g* for 30 min at 4°C. The supernatant was transferred into 12 μL 6X LSB and the pellet re-suspended in 72 μL of 1X LSB. Triplicate fractions were run on a 3% to 12% gradient polyacrylamide gel at 150 V for 1 h and stained with Coomassie blue stain. Gels were scanned using Licor’s Odyssey® Infrared Imaging System and band density calculated with Odyssey Software v2.1. Full-length human dystrophin was used as a control at both 0.3- and 0.5-mg/mL concentrations and did not show any significant difference in percent aggregation between the different concentrations (17.6% ± 7.39 and 17.4% ± 7.30, respectively).

### Circular dichroism

Each purified protein was centrifuged at 14,000 × *g* for 10 min at 4°C and the supernatant diluted to 0.5 mg/mL (for gene therapy and utrophin proteins) or 0.3 mg/mL (for exon-skipped and full-length dystrophins) using PBS. Absorption spectra were acquired with a Jasco J-815 spectropolarimeter, initially at 20°C as controlled by a Peltier device, from 200- to 260-nm wavelength. Spectra were then acquired at 1°C temperature intervals from 20°C to 90°C and the characteristic ellipticity at alpha-helical wavelength (θ_222_) recorded. Molar ellipticity, [θ], was calculated using the following equation: [θ] = θ / (10 × *c* × *l*) where *c* is the molar concentration of the sample (mole/L) and *l* is the path length in cm. Molar ellipticity (with units of degrees, centimeter squared per decimole) was plotted against wavelength for the circular dichroism (CD) spectra. Ellipticity at 222 nm (θ_222_) was normalized, plotted against temperature, and fit by regression analysis in Sigma Plot (Systat Software, Inc., San Jose, CA, USA) using equations for two-state or three-state unfolding [30].

### Differential scanning fluorimetry

Our method closely followed that described by Niesen *et al*. [31]. Briefly, the fluorescent dye SYPRO Orange (#S6650, Life Technologies™, Carlsbad, CA, USA) was incubated at a ratio of 1:1,000 (*w*/*w*) with 0.5 mg/mL (or 0.3 mg/mL for exon-skipped proteins) of purified protein in PBS. The dye/protein solution was aliquoted into 50-μL technical triplicates, and an emission of 610 nm was measured in a real-time PCR instrument (iCycler, Bio-Rad, Hercules, CA, USA) as the temperature was increased from 20°C to 90°C at 1°C temperature intervals. The fluorescence was normalized from 0 to 1, plotted against temperature, and fit by regression analysis in Sigma Plot (Systat Software, Inc., San Jose, CA, USA) using an equation for a two-state unfolding model [30].

### Statistical analysis

Data for percentage aggregation and melting temperatures of single-transition melt curves from CD and differential scanning fluorimetry (DSF) were analyzed using a one-way analysis of variance (ANOVA) with Tukey’s *post hoc* test in Prism software (GraphPad, San Diego, CA, USA), all compared to full-length human dystrophin.

## Results

### Basis for choice of recombinant proteins and gel analysis

**Figure 1:**
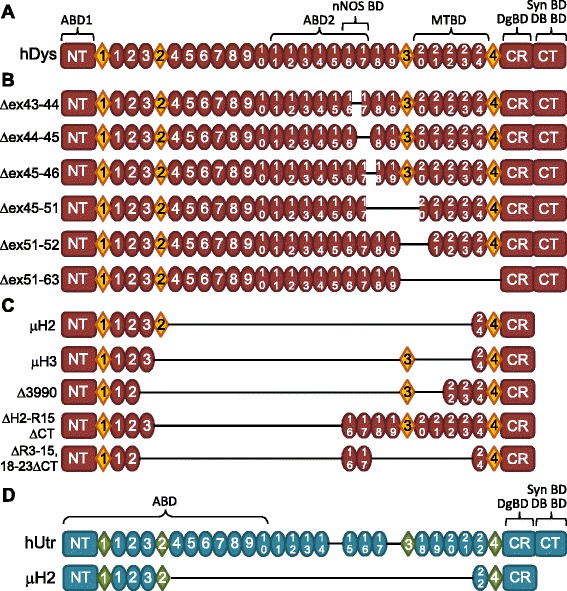
Dystrophin and utrophin constructs analyzed. **(A)** Diagram of full-length human dystrophin (hDys); NT - N-terminus, CR - cysteine-rich domain, CT - C-terminus, circles - spectrin-like repeats, diamonds - unstructured ‘hinge’ regions, ABD1/2 - actin binding domains, nNOS BD - neuronal nitric oxide synthase binding domain, MTBD - microtubule binding domain, DgBD - dystroglycan binding domain, Syn BD - syntrophin binding domain, DB BD - dystrobrevin binding domain. **(B)** Diagrams of exon-skipped human dystrophin constructs analyzed. **(C)** Diagrams of gene therapy human dystrophins analyzed. **(D)** Diagrams of full-length human utrophin (hUtr) and a micro-utrophin (μH2 hUtr) analyzed.

The choice of constructs to analyze was based on current models for exon-skipping and gene therapy in pre-clinical testing and clinical studies (Figure 1). The exon-45-skipped and exon-51-skipped dystrophins were analyzed because they could potentially treat 8% and 13% of DMD patients, respectively [16], and ASO and morpholino drugs targeting these exons are currently in clinical trials [32,33]. Therefore, we expressed a subset of exon-45- and exon-51-skipped human dystrophins: Δex44-45, Δex45-46, Δex45-51, Δex51-52, and Δex51-63, as well as Δex43-44, a control deletion that has been previously speculated to cause decreased stability [34]. While we initially attempted to generate a larger array of exon-51-skipped constructs, we analyzed the three (Δex45-51, Δex51-52, and Δex51-63) that yielded products in the first stages of cloning.

The gene therapy constructs μH2 human dystrophin (hDys) and μH3 hDys contain spectrin-like repeats (SLRs) 1 to 3 and 24 with hinge 2 or hinge 3, respectively. These constructs have been shown to ameliorate the dystrophic phenotype in *mdx* mice [27], and μH2 also showed significant expression with muscle improvement in the GRMD dog model of DMD [35,36]. The Δ3990 hDys construct corresponds to the AAV-delivered micro-dystrophin used in a clinical trial that reported minimal recombinant dystrophin expression associated with a strong immune response to dystrophin [20,37]. Constructs ΔH2-R15/ΔCT hDys and ΔR3-15/18-23/ΔCT hDys are miniaturized dystrophins that retain SLRs 16 and 17 necessary for sarcolemmal localization of neuronal nitric oxide synthase (nNOS) [38,39]. Full-length human utrophin (hUtr) and a micro-utrophin (μH2 hUtr), homologous to μH2 hDys, correspond to constructs that are under investigation for gene-, cell-, and protein-based therapies [25,26,28,40].

**Figure 2:**
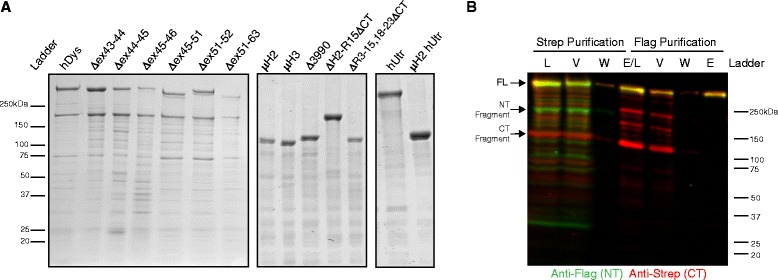
Gel analysis of purified recombinant proteins. **(A)** Representative Coomassie-stained gels with 5 μg of exon-skipped dystrophins, gene therapy dystrophins, and utrophins loaded for comparison. **(B)** Western blot of purification fractions from tandem purification of dual-tagged full-length human dystrophin with N-terminal (NT) FLAG-tag (green channel) and C-terminal (CT) Strep-tag (red channel); fractions from Strep affinity purification and FLAG affinity purifications: load (L), void (V), wash (W), and elute (E). hDys - human dystrophin, hUtr - human utrophin.

While gel analysis of the FLAG-affinity-purified recombinant proteins revealed a predominant band of the expected molecular weight for each purified dystrophin gene therapy and utrophin constructs, full-length and exon-skipped human dystrophins exhibited a near-stoichiometric contaminating fragment at approximately 230 kDa (Figure 2A, left panel) that was not previously observed in preparations of full-length mouse dystrophin [21] and was not present in gene therapy or utrophin preparations (Figure 2A, middle and right panels). To identify the contaminating fragment, we generated and expressed a dual-tagged, full-length human dystrophin containing a C-terminal Twin-Strep-tag® (IBA Life Sciences, Göttingen, Germany) in addition to the N-terminal FLAG-tag. Western blot analysis after tandem affinity purification of the dually tagged dystrophin revealed that the near-stoichiometric, approximately 230-kDa contaminating fragment present in the FLAG-affinity-purified samples was an N-terminal fragment (green band, Figure 2B) and was mostly likely caused by a cleavage from a protease in our expression system. The cleavage event was further confirmed by the presence of the corresponding C-terminal fragment in the load and elution fractions of the Strep-tag purification (red band, Figure 2B). The absence of the contaminating N-terminal fragment in constructs with deletions preceding repeat 16 combined with its presence in constructs with deletions after repeat 16 suggests that the proteolytic cleavage site resides within repeat 14 or 15, which would yield the predicted N-terminal fragment of approximately 230 kDa. While the tandem purification was successful in identifying the contaminating fragments and a more purified full-length dystrophin was recovered (yellow band, Figure 2B), the resulting yield was not sufficient to support the planned biochemical or biophysical analyses. Therefore, single FLAG-affinity-purified proteins were used in all subsequent analyses.

### Protein aggregation

**Figure 3:**
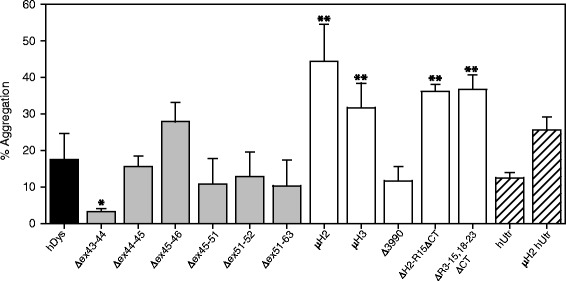
Analysis of protein aggregation by high-speed sedimentation. Quantification of high-speed sedimentation assay of supernatant (*S*) and pellet (*P*) fractions where % aggregation = *S*/(*S* + *P*); full-length human dystrophin (hDys) in black bar, exon-skipped dystrophins in gray bars, gene therapy dystrophins in white bars, and utrophins in lined bars; *P < 0.05, **P < 0.0001 using ANOVA statistical analysis compared to full-length human dystrophin. hUtr - human utrophin.

High-speed sedimentation is a facile *in vitro* technique to quantify aggregation of purified proteins. Full-length human dystrophin exhibited 17.6% aggregation (Figure 3, Table 1), which is similar to the 14% aggregation previously reported for full-length mouse dystrophin [21]. Exon-skipped dystrophin proteins did not show significant increases in aggregation relative to full-length human dystrophin; however, Δex43-44 showed a significant decrease in aggregation (gray bars, Figure 3). Four of the five gene therapy proteins showed a significant increase, with percent aggregation ranging from 31.7% to 44.4% (white bars, Figure 3, Table 1). Interestingly, the Δ3990 protein was the only gene therapy construct that exhibited protein aggregation congruent with full-length dystrophin. Similar to the exon-skipped dystrophins, utrophin and micro-utrophin did not vary significantly from full-length dystrophin aggregation (lined bars, Figure 3).

**Table 1:** **Biophysical properties of human dystrophin and utrophin constructs**

**Construct**	**Molecular weight (kDa)**	**CD Tm1 (°C)**	**CD Tm2 (°C)**	**DSF Tm (°C)**	**% Aggregation**
hDys	427	48.1 ± 1.17	-	45.7 ± 2.62	17.6 ± 7.11
Δex43-44	414	46.7 ± 0.70	-	44.3 ± 0.35	3.4 ± 0.73*
Δex44-45	413	51.7 ± 1.51	-	48.1 ± 1.54	15.7 ± 2.83
Δex45-46	414	48.4 ± 2.38	-	46.0 ± 2.60	27.9 ± 5.28
Δex45-51	385	47.9 ± 4.07	-	44.6 ± 1.63	10.9 ± 7.00
Δex51-52	414	47.2 ± 0.87	-	43.8 ± 0.31	12.9 ± 6.70
Δex51-63	351	48.6 ± 3.08	-	43.7 ± 1.15	10.4 ± 7.11
μH2	139	47.3 ± 1.25	85.1 ± 4.14	46.0 ± 1.28	44.4 ± 10.10**
μH3	137	49.5 ± 1.78	79.0 ± 2.82	45.8 ± 0.86	31.7 ± 6.72**
Δ3990	154	56.1 ± 1.97**	-	50.4 ± 1.91*	11.7 ± 3.97
ΔH2-R15ΔCT	242	53.7 ± 1.51	72.9 ± 4.11	49.8 ± 0.54*	36.2 ± 1.95**
ΔR3-15,18-23 ΔCT	146	45.8 ± 2.96	-	47.3 ± 0.07	36.7 ± 3.96**
hUtr	399	46.2 ± 1.42	-	43.4 ± 0.36	12.5 ± 1.40
μH2 hUtr	138	47.0 ± 1.16	-	42.9 ± 0.25	25.6 ± 3.58

All values are mean values of at least three experiments with standard deviations. CD T_m_1 and T_m_2: circular dichroism melting temperatures; DSF T_m_: differential scanning fluorimetry melting temperatures. *P < 0.05; **P < 0.0001 using ANOVA statistical analysis compared to full-length human dystrophin (hDys). hUtr – human utrophin.

### Assessment of secondary structure unfolding

**Figure 4:**
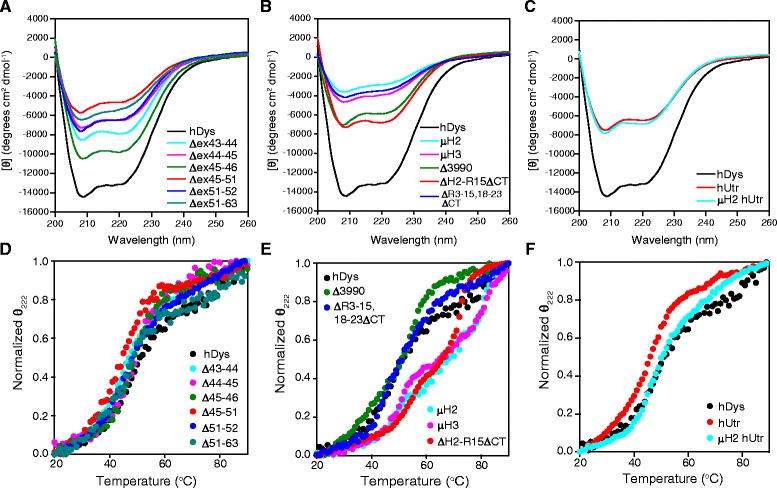
Spectra and melt curves obtained by circular dichroism spectroscopy. **(A-C)** Circular dichroism absorption spectra from 200 to 260 nm for exon-skipped dystrophins **(A)**, gene therapy constructs **(B)**, and utrophins **(C)**. Molar ellipticity [θ], with units of degrees centimeter squared per decimole, was calculated as θ / (10 × *c* × *l*) where *c* is the molar concentration of the sample (mole/L) and *l* is the path length in cm. **(D-F)** CD absorption spectra monitored at 222 nm from 20°C to 90°C for exon-skipped dystrophins **(D)**, gene therapy dystrophins **(E)**, and utrophins **(F**). Melt curves were normalized to θ_222_ from 0 to 1 fraction unfolded and a representative curve plotted. See Table 1 for melting temperatures (CD T_m_1 and T_m_2). hDys - human dystrophin, hUtr - human utrophin.

To assess secondary structure and protein unfolding, we analyzed the purified proteins by CD spectroscopy. All of the constructs exhibited CD spectra characteristic of proteins with high alpha-helical content and minima at 208 and 222 nm (Figure 4A,B,C). As the temperature was increased, loss of secondary structure (or unfolding) was monitored at 222 nm to generate melt curves (Figure 4D,E,F) with a calculated melting temperature or temperatures (Table 1). Full-length human dystrophin exhibited a single-transition melt curve with a melting temperature of 48.1°C (Figure 4, Table 1), which is in contrast to the 59.6°C melting temperature reported for full-length mouse dystrophin [21]. Upon closer inspection of the melt curves from the previous report, it is apparent that full-length mouse dystrophin exhibited an additional melting transition similar to the dystrophin isoform Dp260 [22], a property that is absent in CD melt curves of human dystrophin. Exon-skipped dystrophins exhibited single transitions all with comparable melting temperatures to full-length dystrophin (Figure 4D, Table 1). Two of the gene therapy constructs, Δ3990 and ΔR3-15/18-23/ΔCT, also displayed a single transition, but Δ3990 had a significantly higher melting temperature of 56.1°C. However, the other gene therapy constructs displayed a second transition with two calculated melting temperatures ranging from 47°C to 85°C (Figure 4E, Table 1). This indicates that the protein is either not unfolding cooperatively or is composed of two populations of folded and unfolded states. Full-length human utrophin and μH2 hUtr displayed single-transition melt curves with melting temperatures of 46.2°C and 47°C, respectively (Figure 4F, Table 1). These values are not significantly different from full-length dystrophin and are consistent with previously reported melting temperatures for mouse full-length and micro-mouse utrophins [22].

### Assessment of tertiary structure unfolding

**Figure 5:**
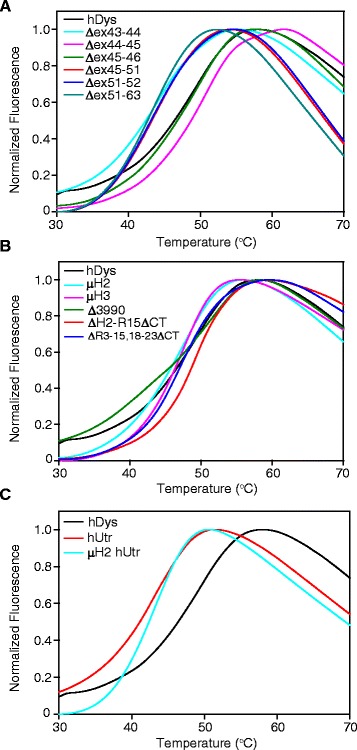
Melt curves obtained by differential scanning fluorimetry. Differential scanning fluorimetry (DSF) melt curves for exon-skipped dystrophins **(A)**, gene therapy dystrophins **(B)**, and utrophins **(C)**. Fluorescence was monitored at 610 nm from 20°C to 90°C, and normalized from 0 to 1 fraction unfolded and a representative curve plotted. See Table 1 for melting temperatures (DSF T_m_). hDys - human dystrophin, hUtr - human utrophin.

DSF utilizes a fluorescent dye that increases its fluorescence emission upon binding to hydrophobic moieties in proteins, which become more exposed and accessible as a protein unfolds during thermal denaturation [31]. Like CD, DSF can be used to obtain protein melt curves, but unlike CD, the signal measured reflects changes in tertiary structure rather than secondary structure. By DSF analysis, full-length human dystrophin displayed a melt curve with a single transition at 45.7°C (Figure 5, Table 1). This temperature was lower than that seen in CD, consistent with the concept that tertiary structure will be lost before secondary structure. Exon-skipped dystrophins displayed single transitions with similar melting temperatures to full-length dystrophin ranging from 43.7°C to 48.1°C (Figure 5A, Table 1). In contrast to the CD data, all of the gene therapy constructs exhibited single transitions with melting temperatures ranging from 45.8°C to 50.4°C (Figure 5B, Table 1). Both Δ3990 and ΔH2-R15/ΔCT hDys had significantly right-shifted melt curves from full-length dystrophin with melting temperatures of 50.4°C and 49.8°C, respectively. Full-length and micro-utrophin both showed a left-shifted melt curve with melting temperatures of 43.4°C and 42.9°C, respectively, but like exon-skipped dystrophins, these values are not significantly different from full-length dystrophin (Figure 5C, Table 1).

## Discussion

In this study, we have analyzed the biophysical properties of several therapeutically relevant, internally truncated dystrophins and utrophins. Therapies that produce internally deleted dystrophins are based on observations that patients with the milder BMD can harbor large deletions in the central rod domain. In addition to conferring elasticity or flexibility to dystrophin [5,41,42], it is known that the central rod domain encodes a second actin binding domain [6,43,44], as well as domains for localizing nNOS to the sarcolemma [38,39], for *in vitro* binding to phospholipids [45,46], intermediate filaments [9], and microtubules [11,12].

The biophysical properties of individual and tandem repeats of the rod domain have been extensively investigated, and these findings demonstrate a wide range of stabilities [45-48], whereas full-length dystrophin has remarkable cooperative stability [21]. Additionally, there is evidence that certain internal deletions of the central rod domain are associated with increased protein aggregation and instability [22]. Together, these studies suggest that the stability of individual or tandem repeat fragments does not necessarily reflect that of larger fragments or full-length dystrophin proteins and that protein stability of individual regions within dystrophin is context dependent.

Several groups have investigated the biophysical consequences of exon-skipping on dystrophin fragments within the central rod domain, particularly those spanning exons 43 to 51 [34,49-51]. For constructs skipping exon 51, they concluded that some protein fragments are more stable than others. Our results, however, suggest that for dystrophins skipped around exon 51, there is little measureable change in stability *in vitro* between exon-skipped proteins and full-length human dystrophin. This conclusion is consistent with the conclusions of a recent report that assessed the structural differences and stability profiles of human dystrophin fragments with deletions around exon 51 [52]. They found that while there were marked structural differences between the different deletion fragments, the stability was not significantly affected.

For the common, out-of-frame deletion of exon 45 (Δ45) in patients, exon-skipping therapies are being designed to either delete exon 44 (Δex44-45) or exon 46 (Δex45-46) to correct the reading frame. Based on another recent report, it was speculated that the Δex45-46 dystrophin protein might be highly unstable because this in-frame deletion is associated with the more severe DMD phenotype in patients [53], and therefore, a Δex44-45-skipping strategy would be more beneficial. However, based on our *in vitro* data, there does not appear to be any significant difference in stability between Δex44-45 and Δex45-46 and full-length dystrophin proteins. These different conclusions from the clinical and *in vitro* studies indicate that the source of pathogenesis from the exon 45 to 46 deletion may not depend on the stability of the resulting protein but, perhaps, is caused by a regulatory or functional perturbation.

Interestingly, *all* of the exon-skipped dystrophins evaluated in our study displayed *in vitro* stabilities congruent with full-length dystrophin, including the Δex43-44 protein that exhibited decreased stability when previously evaluated in the context of a smaller recombinant fragment encompassing SLRs 16 to 18 [34]. Because dystrophy-causing missense mutations also cause less dramatic instability in full-length dystrophin compared to small fragments [21,54], it seems possible that long-range and cooperative intra-protein communication may serve to buffer dystrophin against the destabilizing effects of sequence changes and deletions.

We also expressed internally deleted human micro-dystrophins that are currently under investigation for gene therapy. We showed that several of these constructs have significantly different stability compared to the full-length human dystrophin protein. Both micro-dystrophins μH2 hDys and μH3 hDys displayed increased aggregation and additional melting transitions upon secondary structure unfolding. One of the sarcolemmal nNOS-localizing constructs, ΔH2-R15/ΔCT hDys, displayed similar behavior. Interestingly, the more truncated nNOS-localizing construct, ΔR3-15/18-23/ΔCT, exhibited a single melting transition similar to full-length dystrophin but also increased aggregation. These data suggest that the *in vitro* stability of dystrophin gene therapy constructs may be dependent on the stability of the non-native junction created by the internal deletion. The Δ3990 hDys construct was the only gene therapy construct that exhibited wild-type aggregation, and it displayed significantly increased melting temperatures for both CD and DSF, suggesting that the Δ3990 protein is *more* stable than full-length dystrophin. However, a small clinical trial for AAV-mediated delivery of the Δ3990 was not successful [37].

Utrophin-replacement therapies are also currently under investigation; therefore, we analyzed the stability of full-length human utrophin and a micro-utrophin. Our previous study demonstrated that mouse utrophin is a highly stable protein that does not appear to be sensitive to terminal truncation or internal deletion [22]. Consistent with these results, our data showed that both full-length and micro-utrophin have similar protein aggregation to dystrophin and maintain melting temperatures that are not significantly different from full-length human dystrophin. Investigation of utrophin as a dystrophin substitute therapy for DMD [25,26,28,40] is based on its ability to rescue most phenotypes of the *mdx* mouse when transgenically overexpressed [55] and also the positive correlation between increased utrophin expression with improved prognosis in a small cohort of DMD patients [56]. Utrophin maintains some functional properties of dystrophin, such as forming an analogous utrophin-glycoprotein complex [57] and binding actin filaments [58], but lacks the ability to localize nNOS to the sarcolemma [39,59] and organize the sub-sarcolemmal microtubule lattice [12]. While utrophin cannot completely substitute for dystrophin in terms of protein-protein interactions, our results suggest that utrophin and micro-utrophin proteins are appealing as therapeutic targets in terms of protein stability, especially when compared to dystrophin gene therapy proteins.

While the recombinant dystrophin and utrophin proteins used in this study were expressed in a eukaryotic cellular environment, their purification and characterization in more simple buffers leaves open the possibility that they exhibit unfolding and aggregation properties *in vitro* that are different from how they behave in the complex environment of a mammalian muscle cell. It will therefore be important to develop both cell- and tissue-based model systems to better understand how deletions in dystrophins and utrophins affect stability *in vivo*.

## Conclusions

Our *in vitro* analysis of the biophysical consequences of internal deletion on dystrophin and utrophin suggests that dystrophin stability is context-dependent: relatively unaffected by small deletions at natural exon boundaries but sensitive to larger and more complex rearrangements from deletions present in gene therapy constructs. In contrast, utrophin maintains uniform stability despite large internal deletion. Moreover, our results also highlight the need to better understand how differences in protein stability *in vitro* translate to therapeutic efficacy *in vivo*.

## Abbreviations

AAV: adeno-associated virus
ASO: antisense oligonucleotide
BMD: Becker muscular dystrophy
CD: circular dichroism
CT: C-terminal
DGC: dystrophin-glycoprotein complex
DMD: Duchenne muscular dystrophy
DSF: differential scanning fluorimetry
GRMD: golden retriever muscular dystrophy
hDys: human dystrophin
hUtr: human utrophin
nNOS: neuronal nitric oxide synthase
NT: N-terminal
PMO: phosphorodiamidate morpholino oligomer
SLRs: spectrin-like repeats
T_m_: melting temperature
